# Cell Cycle-Dependent Mobility of Cdc45 Determined *in vivo* by Fluorescence Correlation Spectroscopy

**DOI:** 10.1371/journal.pone.0035537

**Published:** 2012-04-19

**Authors:** Ronan Broderick, Sivaramakrishnan Ramadurai, Katalin Tóth, Denisio M. Togashi, Alan G. Ryder, Jörg Langowski, Heinz Peter Nasheuer

**Affiliations:** 1 Systems Biology Ireland and Centre for Chromosome Biology, School of Natural Sciences, National University of Ireland Galway, Galway, Ireland; 2 Nanoscale Biophotonics Laboratory, School of Chemistry, National University of Ireland Galway, Galway, Ireland; 3 Biophysics of Macromolecules, German Cancer Research Center (DKFZ), Heidelberg, Germany; Texas A&M University, United States of America

## Abstract

Eukaryotic DNA replication is a dynamic process requiring the co-operation of specific replication proteins. We measured the mobility of eGFP-Cdc45 by Fluorescence Correlation Spectroscopy (FCS) *in vivo* in asynchronous cells and in cells synchronized at the G1/S transition and during S phase. Our data show that eGFP-Cdc45 mobility is faster in G1/S transition compared to S phase suggesting that Cdc45 is part of larger protein complex formed in S phase. Furthermore, the size of complexes containing Cdc45 was estimated in asynchronous, G1/S and S phase-synchronized cells using gel filtration chromatography; these findings complemented the *in vivo* FCS data. Analysis of the mobility of eGFP-Cdc45 and the size of complexes containing Cdc45 and eGFP-Cdc45 after UVC-mediated DNA damage revealed no significant changes in diffusion rates and complex sizes using FCS and gel filtration chromatography analyses. This suggests that after UV-damage, Cdc45 is still present in a large multi-protein complex and that its mobility within living cells is consistently similar following UVC-mediated DNA damage.

## Introduction

Duplication of chromosomal DNA is an essential process for both normal cell division and to maintain stability of the genome [1]. Replication of damaged DNA or errors in DNA replication can lead to genetic mutation, with accumulated mutations leading to diseases such as cancer [1]. In human cells, accurate duplication of the genome is carried out by the “replisome progression complex” (RPC), a large multi-subunit complex consisting of replication proteins. These proteins work in concert at different stages of the cell cycle to facilitate DNA replication [2], [3], [4], [5], [6], [7]. Eukaryotic DNA replication begins with the binding of the multi-subunit origin recognition complex (ORC) to the origins of replication at the early G1 phase of the cell cycle [8], [9]. This allows the binding of additional proteins such as Cdc6 (cell division cycle protein 6) and Cdt1 (Cdc10-dependent target) to ORC mediating the loading of the Mcm2–7 (mini-chromosome maintenance) complex to chromatin, forming the pre-replicative complex (preRC) [8], [9]. Activation of the preRC is mediated by CDKs (cyclin-dependent kinases) and DDK (Dbf4-dependent kinase) to allow the binding of Cdc45 and the GINS (go-ichi-ni-san (five-one-two-three)) complex to the Mcm2–7 [8], [9], [10]. This activation of the helicase function of Mcm2–7 allows the formation of a larger multi-subunit protein machinery required for the elongation phase of DNA replication [10], [11] and of single-stranded DNA, which is coated by RPA (replication protein A). DNA polymerase α-primase (Pol-prim) synthesizes the first RNA primer for DNA replication in the origin of replication, which is elongated by its DNA polymerase activity. The RNA–DNA is recognized by RFC (replication factor C), which loads PCNA (proliferating-cell nuclear antigen) [8], [9]. RFC and PCNA, together with RPA, allow a polymerase switch from Pol-prim to Pol (DNA polymerase) ε or *δ*, synthesizing the bulk DNA synthesis on the leading strand and lagging strand, respectively [8], [9].

Cdc45 protein has a key function in the initiation and elongation phases of DNA replication [12] and recent data indicated that Cdc45 is part of the CMG (Cdc45 Mcm2–7 GINS) complex formed at the onset of S phase, progressing to a larger RPC complex present at the elongation phase of DNA replication [13]. Protein-protein interaction studies showed that human Cdc45 interacts with Mcm5, Mcm7 and members of the GINS complex as well as with Pols δ and ε in S phase cells [4]. The current model suggests that in the RPC, Cdc45 forms a molecular bridge between the helicase and DNA polymerase components of the complex [7]. Cdc45 is also the target of a Chk1-mediated intra-S-phase checkpoint [14].

Changes in the overall size of protein complexes containing Cdc45 after activation of the intra-S-phase checkpoint have not been examined and the question remains whether Cdc45 remains as part of the RPC after activation of this checkpoint. Biochemical data demonstrate the role of Cdc45 in initiation and elongation phases of DNA replication, but no *in vivo* data exists to elucidate how Cdc45 is regulated inside cells as part of a multi-protein complex [7]. To shed light on this function, we used Fluorescence Correlation Spectroscopy (FCS) to examine the dynamics of Cdc45 in living cells. FCS is a proven technique to measure mobility of fluorescent molecules *in vivo* by analyzing the temporal fluorescence fluctuations arising from molecules diffusing through a femto-liter detection volume [15], [16], [17], [18], [19], [20], [21], [22]. The small detection volume may be obtained by the use of confocal optics [23]. Typical concentrations of fluorescently tagged molecules in FCS are in the nanomolar range, corresponding to one or a few molecules simultaneously present in the observation volume. These low intracellular protein concentrations pose a limit for *in vivo* FCS measurements, as does the heterogeneity of the cellular environment e.g., movement of organelles and of the entire cell [20]. Furthermore, the autofluorescent protein tag must exhibit a high photostability (such as eGFP), to avoid photobleaching on the time scale of the measurement. Recently, we used FCS to study dynamics of RPA in living cells [24]. Here, we measured the mobility of eGFP-Cdc45 by FCS *in vivo* in asynchronous cells and in cells synchronized at the G1/S transition and during S phase. Our data show that eGFP-Cdc45 moves faster at the G1/S transition than during S phase. Furthermore, the size of protein complexes containing endogenous Cdc45 and eGFP-Cdc45 was estimated for the same cell cycle stages *in vitro* by lysis in a low stringency buffer and gel-filtration chromatography. These data show that eGFP-Cdc45 is part of a multi-protein complex at the G1/S transition and of a very large complex in S phase, which complements the FCS studies obtained *in vivo*. Furthermore, the mobility of eGFP-Cdc45 and the size of complexes containing Cdc45 and eGFP-Cdc45 were studied, following UVC-mediated DNA damage using FCS and gel-filtration chromatography. Surprisingly, both techniques did not reveal significant changes in the mobility and complex sizes. This suggests that after UV-damage Cdc45 is still present in large multi-protein complexes.

## Results

### Generation of HeLa S3 cells stably expressing eGFP-Cdc45

**Figure 1:**
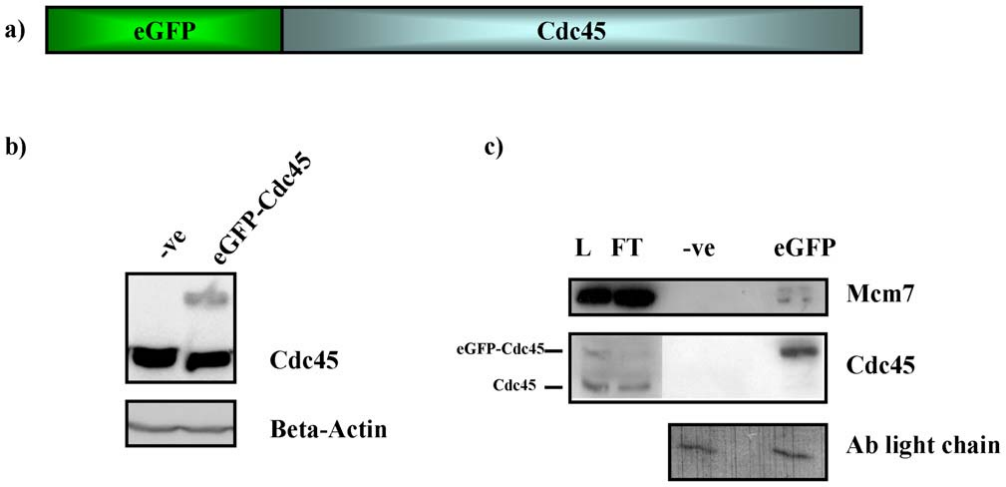
HeLa S3 cells stably expressing eGFP-Cdc45. Panel a, schematic diagram of eGFP-Cdc45 protein encoded by Cdc45L ORF cloned into pIC113gw vector. Panel b, total cell extract HeLaS3 cells (-ve) and Hela S3 cells stably expressing eGFP-Cdc45 (eGFP-Cdc45) normalized for protein content and analysed by western blotting using antibodies raised against Cdc45 and β-Actin, which serves as a loading control. Panel c, western blot analysis of immunoprecipitation of eGFP-Cdc45 using GFP-Trap IP from HeLa S3 cells transiently expressing eGFP-Cdc45. Verification of purification of eGFP-Cdc45 and co-immunoprecipitation of Mcm7 was carried out using antibodies raised against Mcm7 and Cdc45. Input (L), unbound (FT), mock IP (-ve) and IP from cells expressing eGFP-Cdc45 (eGFP) indicate yield of the IP and co-immunoprecipiation. Antibody light chain acts as a loading control.

HeLa S3 cells stably expressing N-terminally tagged eGFP-Cdc45 were generated to analyse Cdc45 in living cells (Figure 1a). This cell line was generated by transfection of a plasmid coding for eGFP-Cdc45 fusion protein under the control of the CMV promoter followed by selection of G418-resistant cells. G418-resistant cells were seeded in culture at a low density to isolate single clones. Clones were selected, expanded and screened for expression of eGFP-Cdc45 by western blotting. Quantitative western blotting showed that eGFP-Cdc45 is expressed at 25% of endogenous untagged Cdc45 in these cells (Figure 1b). In this analysis the signal for eGFP-Cdc45 was compared to the endogenous Cdc45 signal from the same blot. The latter was arbritarily set to 100%. Since this low level of expression caused problems to detect eGFP-Cdc45 by fluorescence microscopy, eGFP-Cdc45 was also transiently expressed to the same level as the endogenous protein. In this case we observed nucleoplasmic localisation, consistent with earlier immunofluorescence studies using an antibody specific for Cdc45 [4]. In addition, the co-immunoprecipitation of eGFP-Cdc45 with a known interacting protein, in this case Mcm7 [4], [14], was carried out and verified by western blotting (Figure 1c). This indicates that the eGFP-Cdc45 fusion protein interacts with essential components of the DNA replication machinery.

### Chromatin association of Cdc45 in the cell cycle and UVC-mediated DNA damage

**Figure 2:**
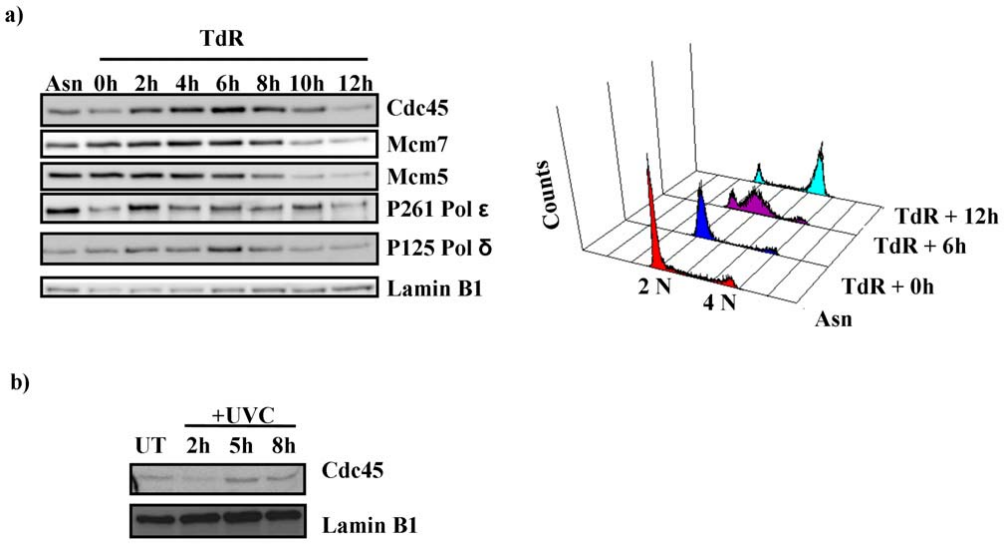
Association of Cdc45 with chromatin synchronized HeLa S3 cells and after DNA damage. Panel a, chromatin-associated lysate from 1×10^6^ Hela S3 cells synchronized at various cell cycle stages by two consecutive thymidine block analysed by western blotting using antibodies raised against Cdc45, Mcm7, Mcm5, Lamin B1, P261 and P125 of Pol ε and *δ*, respectively. The latter serves as a loading control. Asynchronous control cells (Asn) or cells analysed at times ranging from 0 to 12 h following release from the second thymidine block (TdR 0 to TdR 12) were analysed in parallel by FACS. Corresponding FACS profiles for relevant timepoints are also shown. Panel b, western blot of chromatin-associated Cdc45 following UVC treatment. HeLa S3 cells treated with 5 J/m^2^ UVC harvested at indicated timepoints post treatment with untreated cells (UT) acting as a control. Chromatin-associated lysates normalized for protein content were analysed by western blotting using antibodies raised against Cdc45 and Lamin B1, which serves as a loading control.

To verify our method, we tested whether chromatin association of Cdc45 correlated with published data. Chromatin association of Cdc45 as well as Mcm5, Mcm7, p125 and p261 of Pol δ and ε, respectively, were analysed through the cell cycle (Figure 2a). Low levels of Cdc45 associated with chromatin at the G1/S transition whereas Cdc45 maximally bound to chromatin 6 h after the release from the thymidine arrest (Figure 2a left panel) when nearly all cells were in S phase of the cell cycle as determined by FACS analysis (Figure 2a, right panel). The levels of Cdc45 association with chromatin were minimal 12 h after release from thymidine block when most cells were in G2 or M phase. Chromatin association of the Mcm2–7 proteins was already very high at the G1/S transition and remained persistently high throughout S phase, whereas association of replicative DNA polymerases was maximal during S phase.

In addition, chromatin association of Cdc45 was analysed following a sub-lethal dose of UVC (Figure 2b). Two hours after UV treatment, chromatin association of Cdc45 was reduced to about 35% of the level of untreated cells, with recovery to normal levels by 5 h post-treatment, as determined by quantitative western blotting. The level of asynchronously growing cells was arbritrarly set to 100%. Here, Cdc45 has similar chromatin dissociation kinetics when compared to previous data obtained using a low dose of the carcinogen BPDE, which induced a Chk1-dependent dissociation of Cdc45 from the chromatin [14].

### Changes of the diffusion coefficient of eGFP-Cdc45 in HeLa cells during the cell cycle and after UV-mediated DNA damage

The mobility and complex formation of eGFP-Cdc45 during the cell cycle and after UV damage was also studied by FCS in HeLa S3 cells stably expressing eGFP-Cdc45. Fluorescence autocorrelation functions (ACFs) were fitted with a free or anomalous diffusion model with a one- or two-component fit. For the single-component free diffusion model, residuals (quality of 3-dimensional diffusion model fit to experimental data) show a systematic deviation from the fit (Figure S1). The single-component anomalous diffusion model provides a good residual fit with an anomaly parameter α∼0.7±0.1 (standard deviation), however the diffusion time and the calculated diffusion coefficient obtained with this model show a large standard deviation (Figure S2 and Table S1), which is difficult to interpret in our model system. Similarly, a two-component anomalous diffusion model gave a good residual fit, but the anomaly parameter α showed large standard deviations with α being around 1, which corresponds to free diffusion [16] (Figure S3 and Table S2).

**Figure 3:**
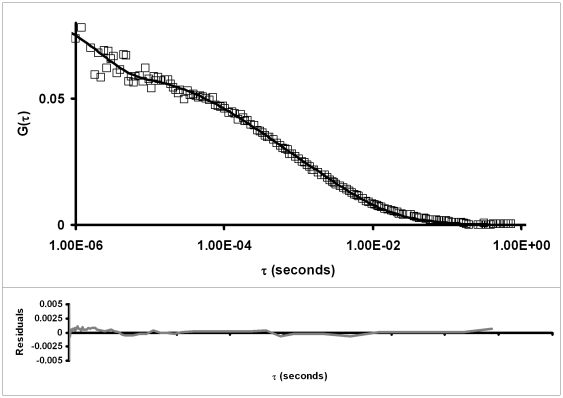
Auto-correlation curves of eGFP-Cdc45. The figure shows typical auto-correlation curves of eGFP-Cdc45 (□) in asynchronous HeLa S3 cells stably expressing eGFP-Cdc45. In the upper panel the solid black line corresponds to a two-component free diffusion model and in the lower panel the gray line is the residual of the fit.

The large deviations in the anomaly parameter α are most likely due to the noise on the ACF and led us to use a two component free diffusion model which gave a very good fit with residuals close to zero (Figure 3, lower panel). The intercellular diffusion coefficient of eGFP and eGFP-Cdc45 analysed with the two-component free diffusion model are summarized in Table 1. The fractions r1 and r2 of the slow and the fast component, were about 40% and 60%, respectively, and remained similar throughout the cell cycle and after UVC treatment. The fast component with the diffusion coefficient (*D*2) was observed in eGFP transfected and eGFP-Cdc45 stably expressing HeLa S3 cells, but not in untransfected HeLa S3 cells. The diffusion times of the fast component were in the range of 30–120 µs, which is in the range of the known non-diffusive component of the ACF due to protonation/deprotonation kinetics of eGFP [25]. The diffusion coefficients *D2* of the fast component were at least one order of magnitude larger than the *D1* values of the slow component. The ratios of the diffusion coefficients of the slow to those of fast component were constant throughout the cell cycle, therefore, we will focus on the slow component for further discussion and interpretation. Hereafter the diffusion coefficient of the slow component will be called as *D*. We determined the *in vivo* diffusion coefficient *D* of eGFP to be 34±4 µm^2^ s^−1^, similar to values found by other labs [18], [20], [26], [27]. The rather large variations among reported values, including our own published values, are most probably due to variations in cell lines, growth conditions, and other biological variations.

**Table 1:** Diffusion coefficient of eGFP-Cdc45 at G1 to S phase transition and in S phase and after UV damage.

	D1 ± SD (µm^2^ s^−1^)	r1	D2 ± SD (µm^2^ s^−1^)	r2
eGFP	34±4.0	0.59±0.17	500±200	0.41±0.17
eGFP-Cdc45	8.2±3.0	0.41±0.17	159±97	0.59±0.17
eGFP-Cdc45 G1/S	9.7±2.0	0.43±0.2	175±340	0.57±0.2
eGFP-Cdc45 S phase	3.6±1.2	0.3±0.1	60±37	0.7±0.1
eGFP-Cdc45 UVC	7.7±3.0	0.49±0.2	116±73	0.51±0.19

Mean and standard deviations obtained from at least 15 cells.

The FCS measurements yielded a number of 20 to 40 (average: 31±10) eGFP-Cdc45 molecules per femtolitre in these stably eGFP-Cdc45-expressing HeLa S3 cells. With a nuclear volume of about 500 to 600 fl for the HeLa S3 cells [24] this would yield 10,000 to 20,000 molecules of eGFP-Cdc45 per cell. Our quantitative western blot experiments shown in Figure 1b revealed that the ratio of eGFP-Cdc45 to endogenous Cdc45 is approximately 1 to 4 resulting in an estimate endogenous Cdc45 number of 40,000 to 80,000 molecules per cell, which is in agreement with previous estimated numbers of Cdc45 molecules per cell being 45,000 [28].

The diffusion coefficient *D* of eGFP-Cdc45 in asynchronous cells shows a broad range, suggesting a large variation in the sizes of the protein complexes containing eGFP-Cdc45 and that the protein may function in a cell cycle dependent manner (Table 1). In cells arrested at the G1/S transition, the diffusion coefficient *D* of eGFP-Cdc45 is comparable to that in asynchronous cells, but shows a smaller standard deviation (Table 1) suggesting less variability in the sizes of eGFP-Cdc45-containing complexes. In S phase, we determined *D* of eGFP-Cdc45 as 3.6 µm^2^ s^−1^ with standard deviations of 1.2 µm^2^ s^−1^ in comparison to *D* of 9.7±2 µm^2^ s^−1^ in G1/S transition phase. The molecular brightness (CPSM) of eGFP-Cdc45 in S phase did not change significantly compared to G1/S transition or to eGFP, excluding higher oligomeric states of eGFP-Cdc45 in S phase (Figure S4) [29]. Estimating sizes of protein complexes in living cells by FCS remains very difficult due to variability in the intracellular environment [18]. Nevertheless, we attempted to estimate the relative molecular masses of the diffusing complexes from the diffusion times as suggested by Brazda and colleagues [19], assuming that the molecule has a spherical shape. To achieve this, we compared the diffusion times of eGFP-Cdc45 and eGFP. The molecular mass of the eGFP-Cdc45-complexes varied from 1 MDa in cells at the G1/S transition to 30 MDa in cells synchronised during S phase of the cell cycle, while the monomeric form of eGFP-Cdc45 weights approximately 95 kDa. After UVC-mediated DNA damage, we observed no significant change in the diffusion coefficient of eGFP-Cdc45. To complement the FCS data generated, we examined the sizes of complexes containing eGFP-Cdc45 and endogenous Cdc45 at different cell cycle stages and after UVC mediated DNA damage by gel filtration chromatography.

### Analysis of Cdc45 containing complexes by gel filtration chromatography

**Figure 4:**
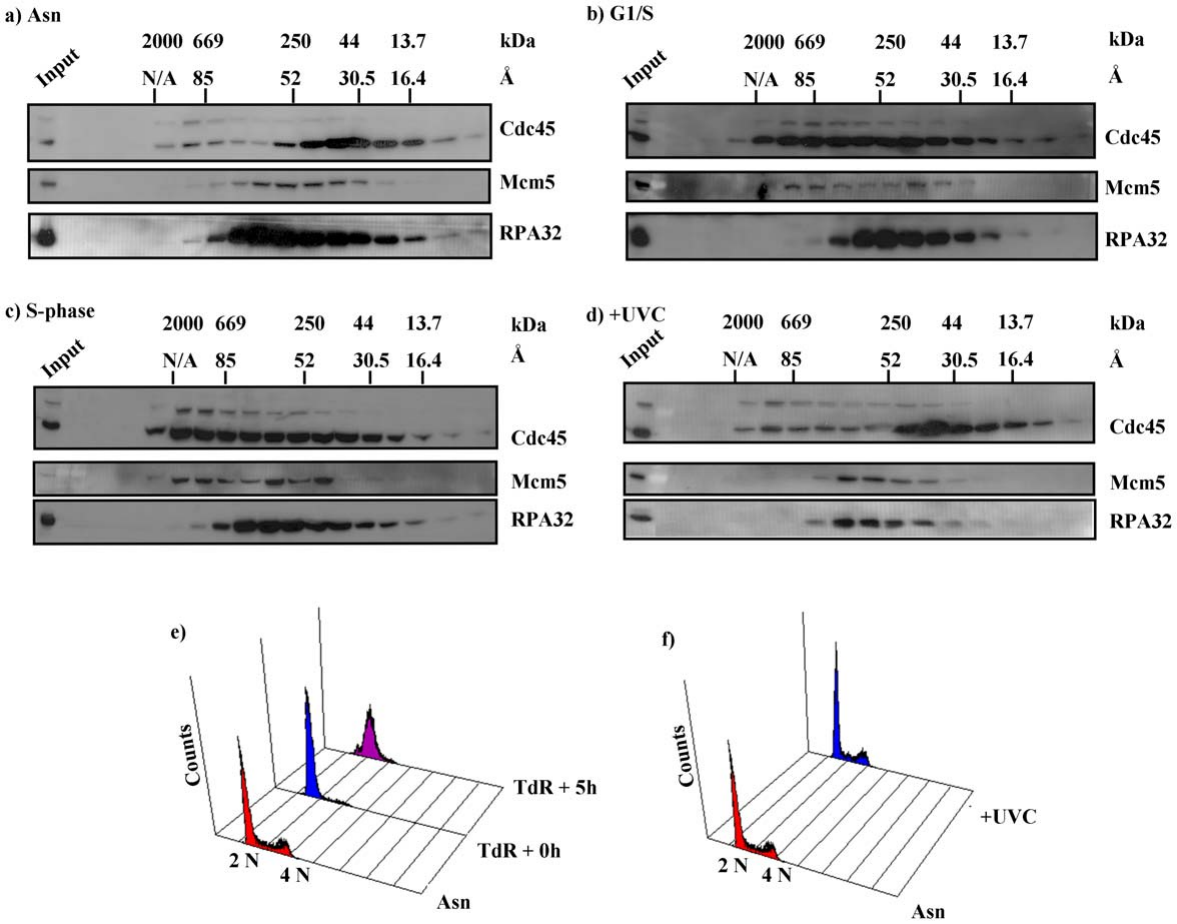
Analysis of the size distribution of Cdc45-containing protein complexes during the cell cycle and after UV damage. Asynchronous (Asn) UVC-treated (+UVC, 5 J/m^2^, 1 h post-treatment), G1/S transition or S phase synchronized HeLa S3 cells stably expressing eGFP-Cdc45 were lysed and normalized for protein content and separated by gel filtration chromatography analysed by western blotting using antibodies raised against Cdc45, Mcm5 and RPA 32. (panels a, b, c, and d, respectively). Theoretical molecular weight (kDa) and Stoke's radius (Å) of protein standards are overlayed. RPA32 acts as a marker for DNA damage response following UVC treatment. FACS analysis is provided for asynchronous (Asn), G1/S transition and S phase synchronized cells (e) and asynchronous cells treated with 5 J/m^2^, 1 h post-treatment (f).

Figure 4 shows examples of western blots of gel filtration profiles. In these experiments we find a mixture of complex sizes, with the most prevalent forms of Cdc45 being an apparently monomeric population and a complex of the same size as the thyroglobulin standard (669 kDa). In asynchronous cells the monomeric Cdc45 is the prominent component, whereas in G1/S synchronized cells we observed a shift of the Cdc45-containing complexes towards the 700 kDa-sized complex (Figure 4, compare panel a with b). In S phase-synchronized cells the size of the Cdc45-containing protein complexes further increased, with the largest complexes approaching 2 MDa (Figure 4a). In these gel filtration experiments endogenous Cdc45 and eGFP-Cdc45 behave identically, taking into account that the monomeric eGFP-Cdc45 is 27 kDa larger than monomeric endogenous Cdc45. Interestingly, as observed in the FCS experiments (Table 1), the complex formation of Cdc45 did not change after treating asynchronous cells with UVC (Figure 4, compare panel a with panel d).

For comparison, Mcm5 protein was analysed in the same fractions of the gel filtration chromatogram as eGFP-Cdc45 in asynchronous, G1/S transition- and S phase-synchronized cells. In contrast to Cdc45 and Mcm5, the heterotrimeric RPA complex shows approximately the same size in asynchronous and synchronized cells in the gel filtration experiments as marked by the second largest subunit of RPA, RPA32 (Figure 4 a to c, lowest panels). Moreover, we found no changes in the overall size of complexes containing Cdc45, Mcm5 and RPA32 after UVC treatment, with phosphorylation of RPA32 observed in UVC treated cells (Figure 4). These findings fit well with gel filtration chromatography analyses of isolated CMG complexes from *Drosophila*, with a size of the CMG complex roughly equal to thyroglobulin (669 kDa) [10]. A separate study in budding yeast estimated the size of RPC, with the CMG complex at its core, to be about 2 MDa in S phase-synchronized extracts [13].

## Discussion

To study the replication protein Cdc45 *in vivo*, we generated HeLa S3 cells that stably expressed eGFP-Cdc45. The expression levels of eGFP-Cdc45 in these cells were approximately 25% of that of endogenous Cdc45. These low levels were particularly suitable for FCS studies. Functionally, we showed that the eGFP-Cdc45 fusion protein behaved identically to endogenous Cdc45 in all biochemical assays [4]. Co-immunoprecipitation of Mcm7 with eGFP-Cdc45 also validated its functionality. Interestingly, little endogenous Cdc45 co-immunoprecipitated with eGFP-Cdc45, suggesting that the eGFP-Cdc45-containing CMG and RPC complexes contain only one molecule of Cdc45 per complex. Transiently expressed eGFP-Cdc45 localized in the nucleoplasm of HeLa S3 cells, similar to previously published localisation data for Cdc45 [4]. Moreover, gel filtration chromatography showed eGFP-Cdc45 in the same fractions as the endogenous protein, suggesting that eGFP-Cdc45 can form the same complexes as endogenous Cdc45.

The mobility of eGFP-Cdc45 was measured by FCS in asynchronous HeLa S3 cells, G1/S transition-, S phase-synchronized cells and in asynchronous cells following UVC-induced DNA damage. The mobility of eGFP-Cdc45 was broadly distributed in asynchronous HeLa S3 cells (Table 1) whereas its mobility in S phase was reduced approximately 3 times compared to cells at the G1/S transition (Table 1). Recent reports showed that fluorescently tagged Cdc20 protein (∼85 kDa) [22] and PLK1 protein (∼93 kDa) [20], which are similar in molecular weight to eGFP-Cdc45, diffuse slowly in living cells. Monomeric Cdc20 protein in living cells moved six times faster than the multiprotein complexed form [22]. Upon mutation, the catalytic activity of PLK1 protein decreased and its mobility increased, reflecting a reduction in complex size [20]. Comparing the diffusion rates of monomeric Cdc20 and eGFP-Cdc45 in living cells suggests that eGFP-Cdc45 is putatively a part of multi-protein complexes which may vary throughout the cell cycle. We estimated the sizes of Cdc45-containing complexes to be about 1 MDa at the G1/S transition and 30 MDa in S phase using FCS mobility data. To corroborate these results, we investigated the sizes of complexes containing both endogenous and eGFP-tagged Cdc45 by gel filtration chromatography. A low stringency lysis buffer was used to solubilise proteins whilst keeping protein complexes intact [30]. Size analysis of eGFP-Cdc45-containing complexes by gel filtration chromatography gave results consistent with similar analyses of isolated CMG complexes from *Drosophila* of an estimated size of about 0.7 MDa [10]. The observation of a larger complex during S phase, approaching 2 MDa, is consistent with gel filtration analyses of proteins from budding yeast, where isolated RPCs incorporating the CMG complex had an estimated molecular mass of about 2 MDa [13]. The hypothesis that these complexes observed in gel filtration chromatography assays are indeed the CMG and RPC is supported by the observation that Mcm5 protein is present in the same fractions as Cdc45 in the G1/S transition and S phase-synchronized cells. The estimated sizes of Cdc45-containing complexes at the G1/S transition were slightly larger in living cells, as determined by FCS, than in the cell extracts determined by gel filtration. A possible explanation is that either proteins other than the Mcm2–7 and the GINS complexes may associate with Cdc45 *in vivo*, that the complexes bind transiently to immobile structures, or that the assumption needs to be reconsidered that the Cdc45 protein complex has a spherical shape. In the latter case, a different model for the molecular shape might yield a molecular mass closer to the CMG core complex.

Even so, the gel filtration data correlate well with the FCS-observed diffusion rates for eGFP-Cdc45, which confirms that the *in vitro* biochemical assay reflects the dynamics of the actual *in vivo* eGFP-Cdc45-containing complexes. As the cell progresses to S phase, Cdc45 as part of the CMG complex forms an active RPC complex [13]. Although both methods showed an increased size of the S phase Cdc45 complexes compared to the G1/S complex, the complex sizes determined by gel filtration and FCS, 2 MDa versus 30 MDa, clearly differ. This finding can be explained in different ways. First, proteins including RPA might have been lost during gel filtration and therefore the size of S phase Cdc45 complex in living cells was underestimated *in vitro*. This is consistent with the findings that the gel filtration carried out did not contain some proteins which are part of RPC, such as RPA. However, the protein diffusion in living cells is governed not only by the size of a complex but also by interactions with stable scaffolds such as chromatin [31], [32]. Therefore, the slower mobility of Cdc45-containing complex might be caused by the Cdc45-containing transiently binding to DNA, chromatin, or other cellular structures as previously discussed for RPA [24].

Analyses of chromatin association of replication proteins presented here support the model that these proteins form functional RPC on chromatin [11]. Our chromatin association data are consistent with reports showing the formation of the CMG complex after the G1/S transition as chromatin association of Mcm2–7 proteins is seen here as well as some chromatin association of Cdc45 [11]. Association of Cdc45 to chromatin at the G1/S transition with maximal association at S phase is consistent with data demonstrating binding of Cdc45-GINS to Mcm2–7 to form the CMG complex in a CDK- and DDK-dependent manner. This would activate the helicase function of the complex at the onset of S phase [10], coinciding with the formation of the fully functional RPC. Maximal association of the elongating DNA polymerases during S phase is consistent with binding of these proteins to the active CMG as well as other factors including Mcm10, Pol-prim, RFC, RPA, Claspin/Timeless/Tipin, and Ctf4/And1 to create the RPC [4], [9], [13], [33], [34]. Following UVC treatment the affinity of Cdc45 for chromatin is reduced between 0 and 2 h post treatment, with a recovery between 2 h and 5 h post-treatment. In addition, phosphorylation of RPA32 is observed in UV-treated samples, indicating the activation of damage response. These data display similar kinetics when compared with previously observed Chk1-dependent reductions in chromatin-association of Cdc45 following treatment of H1299 cells with BPDE [14]. However, FCS and gel filtration chromatography showed no discernable effect on the overall complex size of replisomes containing Cdc45 and on the distribution of eGFP-Cdc45, Mcm5 or RPA32 after UV treatment when compared to the asynchronous control. This lack of change in size of eGFP-Cdc45-containing complexes following UV-mediated DNA damage suggests that intact RPCs containing Cdc45 are present following damage and might be reloaded as such to start DNA replication again. These findings are consistent with a model where RPCs containing Cdc45 undergo a conformational change following UV-mediated damage, but still contain Cdc45. Recent studies demonstrate that the CMG complex can exist in a “locked” form and a more open “notched” form [35], where the gap observed between Cdc45 and the most proximal Mcm2–7 proteins is larger in the “notched” conformation than in the “locked” form. A change in the overall structure or conformation of the RPC complex containing Cdc45 following fork stalling could explain the observed reduction in Cdc45's affinity for chromatin, its affinity for Mcm proteins, and the lack of a significant change in complex size or diffusion rate for eGFP-Cdc45 in living cells after UVC treatment (Table 1). Alternatively, it may well be that the fraction of Cdc45 associated to chromatin in asynchronous cells is too small to be detected by the FCS and gel filtration chromatography assays.

We show that FCS can be used to characterize the mobility and the dynamics of eGFP-Cdc45 *in vivo* in real time. The mobility change of eGFP-Cdc45 and the size of eGFP-Cdc45 and Cdc45-containing complexes as analysed by FCS and gel-filtration suggests that the association state of the protein is different at the G1/S transition and during S phase. In contrast, we did not observe a significant change in eGFP-Cdc45 mobility or complex size after UVC-induced DNA damage, but did see a reduction in Cdc45-chromatin association after damage; this suggests that large Cdc45 complexes remain stable after removal from chromatin. These observations merit further investigation by biochemical methods and FCS.

## Materials and Methods

### Cell Culture

HeLa S3 cells (ATCC CCL2.2) [4] were cultured in Dulbecco's modified Eagle's medium (Sigma) supplemented with 10% foetal calf serum (Sigma), 100 units/ml penicillin (Lonza) and streptomycin (Lonza). Cells stably expressing eGFP-Cdc45 were selected and cultured in media supplemented with 500 µg/ml G418 sulphate (Lonza). For FCS, HeLa S3 cells that which stably express eGFP-Cdc45 were grown in eight-well chambered cover glass slides (Nunc, Denmark), Dulbecco's modified Eagle's medium (DMEM, GIBCO) without phenol red, with 10% Foetal calf serum (Sigma), 1% L-glutamine (Sigma) at 37°C in a humidified incubator containing 5% CO_2_.

### Antibodies

Antibodies recognizing Cdc45 (C45-3G10-111) [4], p125 of Pol δ (PGD-5E1) [28] and RPA 32 (RBF-4E4) [36], [37]. Anti-Mcm5 antibody was obtained from Prof R. Knippers [38]. Mcm7 antibody was obtained from Neomarkers (47DC141). Antibody raised against β-Actin was obtained from Sigma (A5441). Antibody raised against Lamin β1 was obtained from Abcam (ab16048). P261 Pol ε antibody was obtained from BD Biosciences (#611238).

### Generation of eGFP-Cdc45 plasmid constructs

The ORF of Cdc45 was cloned into the gateway entry vector pENTR3C (Invitrogen) between the BamH1 and EcoRI restriction sites. The ORF was then recombined into a gateway-adapted eGFP vector based on the pIC113 backbone (pIC113gw, a generous gift from Prof. K. Sullivan) using the LR Clonase II enzyme kit (Invitrogen), as described by the manufacturer.

### Fluorescence correlation spectroscopy

FCS measurements were carried out in laboratory-built set up as described in [18]. In short, confocal laser scanning microscopy (CLSM) was performed with a built in FCS module attached via an X-Y galvanometer scanner to an inverted microscope (Olympus IX70, Hamburg, Germany) with an UplanApo/IR 60 X water immersion objective lens (Numerical aperture: 1.2). For *in vivo* imaging and FCS, HeLa S3 cells stably expressing eGFP-Cdc45 were plated into eight-well chambered coverslips (Nunc, Denmark) and measured at 37°C in a 5% CO_2_ humidified atmosphere, in an incubator chamber surrounding the entire microscope. An argon-krypton laser with a 488 nm laser created the fluorescence excitation, and emissions were detected between 515 to 545 nm with avalanche photodiodes (APDs) (SPCM-AQR-13, Perkin-Elmer, Wellesley, USA) after passage through appropriate dichroic mirrors and filters for spectral separation. Home-made control software allowed us to choose points from confocal fluorescence images of nuclei for FCS measurements. The signals coming from the APDs were fed into an ALV-5000/E correlator card (ALV Laser GmbH, Langen, Germany), which accumulated the autocorrelation function in parallel for all time delays and in real time.

Nuclei were imaged using CLSM and 5 random positions were selected. Autocorrelation measurements of 5 runs of 10 seconds each were performed on each measurement point. The laser power was 6 µW; no significant change in count rate due to photo-bleaching was observed. For each dataset, we measured at least 15 cells on different days. Data sets that showed unusual variation or oscillation in the count rates were excluded from our analysis. The setup was calibrated using 20 nM AlexaFluor 488 dye (Molecular Probes) in water as described [18].

### Data analysis

The autocorrelation curves of multiple runs at each individual measured point were fitted by a non-linear fitting using the QuickFit software employing the Marquardt-Levenberg algorithm (http://www.dkfz.de/Macromol/quickfit/). The autocorrelation curves of were fitted with a two diffusional component, triplet correction and a term for eGFP blinking [16], [19].
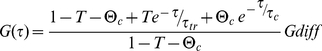
(1)where
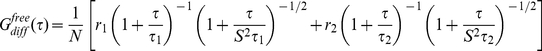
(2)

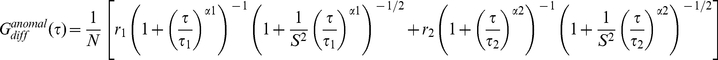
(3)The autocorrelation function with the term accounting for triplet state formation and dark state formation due to protonation (i.e. blinking) and the term accounting for diffusion are (*G_diff_*) were considered when analysing the data [25]. *N* is the average number of fluorescently or tagged molecules in the confocal volume, *τ* is the lag time. In the triplet term *T* denotes the equilibrium molar fraction of flurophores in the triplet state and *τ_tr_* is the triplet life time [39]. The protonation mechanism [25] is characterized by the molecular fraction Θ*_c_* and the correlation time *τ_c_*.

Two diffusion terms were assumed: a slow species with mole fraction *r*_1_ and diffusion time *τ_1_*, and fast species with mole fraction *r*_2_ = 1−*r*_1_ and diffusion time *τ_2_*. *S* denotes the structure factor, which depends on the focal volume and is given by *S* = **Z**_0_/*w*_0_, where **Z**_0_ is the axial and *w*_0_ the lateral dimension of the confocal volume. The anomaly parameter α corresponds to the mechanism of diffusion; α = 1 corresponds to free diffusion while α<1 corresponds to obstructed diffusion [16]. Anomalous diffusion can result from transient binding or molecular crowding [40].

The diffusion time *τ_i_* (i = 1,2) is related to the diffusion coefficient *D* by:
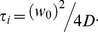
(4)The lateral radius (*w*_0_) at 488 nm excitation was estimated from the diffusion coefficient of Alexa Fluor 488 (20 nM in deionized water). The calculated lateral radius was *w*_0_ = (250±5) nm, taking *D* as 435 µm^2^ s^−1^ at 22.5°C [41].

The change in apparent molecular mass of proteins was calculated based on the Stokes-Einstein equation [19]. Assuming the diffusing species as spheres,
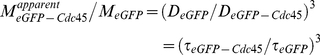
(5)where *M_eGFP_* = 27 kDa is the molecular mass of eGFP, *D* and *τ* correspond to the diffusion coefficient and diffusion time of eGFP-Cdc45 and eGFP as indexed.

### Cell synchronisation

HeLa S3 cells and eGFP-Cdc45-expressing HeLa S3 cells were enriched in late G1 to early S phase by performing two consecutive thymidine blocks as previously described [4]. In short, cells were cultured for 24 h in media without thymidine before addition of 5 mM thymidine for 16 h. Subsequently, cells were washed 2 times with PBS and allowed to grow in DMEM without thymidine for 12 h before a second treatment with 5 mM thymidine for 16 h. After this incubation time, cells synchronized at the G1/S transition were analysed using FCS. Cells were washed with PBS, fresh media was added and 5 h later, FCS measurements were carried out for S phase-synchronized cells.

### Cell lysis and immunoblotting

Total cell lysates were prepared in RIPA buffer (1% Triton X-100, 0.5% deoxycholate, 1% Sodium Dodecyl Sulphate (SDS) in PBS, pH 7.4) supplemented with phosphatase inhibitor cocktail II (Sigma) and ETDA-free protease inhibitor cocktail (Roche Applied Sciences). Lysates for immunoprecipitation were prepared in TGN buffer (50 mM Tris-HCl pH 7.5, 200 mM NaCl, 50 mM sodium β-glycerophosphate, 50 mM Sodium Fluoride, 1% Tween-20, 0.2% NP-40) supplemented with phosphatase inhibitor cocktail II (Sigma) and ETDA free protease inhibitor cocktail (Roche Applied Sciences). Briefly, cells were lysed for 20 min on ice and centrifuged for 10 min at 13,000× g at 4°C. Supernatant fractions were collected and used as input for immunoprecipitation experiments.

Lysates for gel filtration chromatography experiments were prepared in 50 mM Tris-HCL pH 7.4, 250 mM NaCl, 5 mM EGTA, 3 mM MgCl_2_ 0.1% NP-40 supplemented with phosphatase inhibitor cocktail II (Sigma) and ETDA free protease inhibitor cocktail (Roche Applied Sciences). Cells were lysed for 20 min on ice before addition of Benzonase (Sigma, adjusted to 250 U/10^7^ cells) and incubation at 25°C for 30 min to solubilise chromatin associated proteins. Lysates were then clarified by centrifuging for 30 min at 100,000× g at 4°C. Supernatants were collected and used as input for gel filtration chromatography.

Lysates, to analyse chromatin association of various proteins, were prepared in a procedure adapted from Liu and colleagues [14]. In brief, 1×10^7^ HeLa S3 cells were rinsed once in PBS and scraped into a minimal volume of ice cold PBS. Cells were then centrifuged at 1000× g for 2 min and resuspended in 250 µl CSK buffer (10 mM PIPES, pH 6.8, 100 mM NaCl, 300 mM sucrose, 3 mM MgCl_2_, 1 mM EGTA, 1 mM dithiothreitol, 0.1 mM ATP, 1 mM Na_3_VO_4_, 10 mM NaF, and 0.1% Triton X-100) supplemented with EDTA free protease inhibitor cocktail (Roche Applied Sciences) and incubated on ice for 4 min. Lysates were then centrifuged at 1000× g for 2 min, and the supernatant was collected as the triton-soluble fraction. Triton-insoluble pellet was washed once in 1 ml CSK buffer and resuspended in 100 µl CSK buffer. RNase-free DNaseI (Applichem), adjusted to 250 U/10^7^ cells was then added and lysates were incubated for 30 min at 25°C to release chromatin-associated proteins. Lysates were then centrifuged at 4°C and 10,000× g for 10 min and the supernatant fractions collected as chromatin-associated fractions.

Cell extracts were resolved by SDS-PAGE, transferred to PVDF membrane and analysed by western blotting using antibodies raised against specific proteins. Western blots were then probed with horseradish peroxidase-conjugated secondary antibodies (HRP, Jackson Immuno Research) and visualized using the ECL or ECL Plus chemiluminescent solution (GE Healthcare).

### Quantitative western blotting

For quantitative western blotting, images of western blots were acquired using a LAS3000 imaging system and software (Fuji). Images were analysed quantitatively using Multi Guage V2.2 software (Fuji) to determine the relative signal intensities of distinct bands.

### Immunoprecipitation

eGFP-Cdc45 was immunoprecipitated from cells using GFP-Trap® (ChromoTek) and low adhesion tubes (Bioquote Limited). Briefly, 2×10^7^ HeLa S3 cells were transfected with 20 µg plasmid coding for eGFP-Cdc45 protein using Fugene/X-tremeGENE HP transfection reagent (Roche Applied Sciences), harvested 24 h post-transfection and lysed in TGN buffer. Protein A/G agarose resin (Calbiochem) was washed three times in TGN buffer and 5 mg lysate was incubated with this resin for 30 min at 4°C to pre-clear. 20 µl packed GFP-Trap®-A resin was washed three times in TGN buffer and incubated with 5 mg pre-cleared lysate from cells transfected with eGFP-Cdc45 plasmid or from mock-transfected control cells. Lysates were incubated with the resin for 2 h at 4°C and washed four times with 1 ml of TGN buffer. Bound proteins were solubilised by boiling beads in 40 µl of 2× Laemmli buffer and centrifugation at 5,000× g for 5 min. Supernatant fractions were collected and analysed by SDS-PAGE and western blotting.

### Flow cytometry and FACS analysis

1×10^6^ HeLa S3 cells were trypsinized, washed in ice cold PBS and resuspended in 1 ml PBS. Ice cold ethanol was added to a final concentration of 75% to fix samples for flow cytometry. Before FACS analysis samples were centrifuged at 1500× g, the supernatant was removed, and the cell pellet was resuspended in 1 ml propidium iodide with RNAse solution (BD Biosciences) and incubated overnight at 4°C. Flow cytometry was carried out using a FACS CANTO flow cytometer (BD Biosciences) and flow cytometry data was analysed using WinMDI software.

### Gel filtration chromatography

Gel filtration of lysates was carried out using a Superdex 200 PC 3.2/30 column and an Äkta Ettan HPLC machine (Amersham Biosciences) with a 50 µl injection loop. The column was calibrated using a high molecular weight (HMW) gel filtration calibration kit (28-4038-42 GE Healthcare) and 300 µg of lysate prepared for gel filtration chromatography as described above was then loaded onto the column. Fractions of 100 µl volume were collected, snap frozen and lyophilised using a freeze dryer (Labconco). Freeze-dried fractions were then resuspended in 20 µl 1× Laemmli buffer, boiled for 5 min and analysed by SDS-PAGE and western blotting.

## Supporting Information

Figure S1 **Autocorrelation function of eGFP-Cdc45 in asynchronous HeLa S3 cells stably expressing eGFP-Cdc45 fitted to one-component free diffusion model.** (□) corresponds to experimentally determined autocorrelation function. The solid black line is the diffusion model fit and the gray line corresponds to the residual of the fit.
(DOC)

Figure S2 **Autocorrelation function of eGFP-Cdc45 in asynchronous HeLa S3 cells stably expressing eGFP-Cdc45 fitted to one-component anomalous diffusion model.** (□) corresponds to experimentally determined autocorrelation function. The solid black line corresponds to the diffusion model fit and the gray line corresponds to the residual of the fit.
(DOC)

Figure S3 **Autocorrelation function of eGFP-Cdc45 in asynchronous HeLa S3 cells** stably expressing eGFP-Cdc45 fitted to a two-component anomalous diffusion model. (□) corresponds to experimentally determined autocorrelation function. The solid black line corresponds to the diffusion model fit and gray line corresponds to the residual of the fit.
(DOC)

Figure S4 **Molecular brightness (CPSM) of eGFP which was transiently expressed in HeLa S3 cells and from HeLa S3 cells stably expressing eGFP-Cdc45 in different cell cycle stages, in UV treated and Asynchronous cells.** Error bars correspond to standard deviation from at least 15 cells.
(DOC)

Table S1 **Single-component anomalous diffusion model fit to eGFP, eGFP-Cdc45 in different cell cycle stages and UVC treatment.**
(DOC)

Table S2 **Two-component anomalous diffusion model fit to eGFP, eGFP-Cdc45 in different cell cycle stages and following UVC treatment.**
(DOC)
