# Supplementary material for: Cell Cycle-Dependent Mobility of Cdc45 Determined in vivo by Fluorescence Correlation Spectroscopy
Source: PLoS One. 2012 Apr 19;7(4):e35537. doi: 10.1371/journal.pone.0035537 (PMC3334904; doi:10.1371/journal.pone.0035537)
Supplement: Figure S2 — Autocorrelation function of eGFP-Cdc45 in asynchronous HeLa S3 cells stably expressing eGFP-Cdc45 fitted to one-component anomalous diffusion model. (□) corresponds to experimentally determined autocorrelation function. The solid black line corresponds to the diffusion model fit and the gray line corresponds to the residual of the fit. (DOC) [file pone.0035537.s002.doc]

**Figure S2**: Autocorrelation function of eGFP-Cdc45 in asynchronous HeLa S3 cells stably expressing eGFP-Cdc45 fitted to one-component anomalous diffusion model. (□) corresponds to experimentally determined autocorrelation function. The solid black line corresponds to the diffusion model fit and the gray line corresponds to the residual of the fit.
