# Supplementary material for: Cell Cycle-Dependent Mobility of Cdc45 Determined in vivo by Fluorescence Correlation Spectroscopy
Source: PLoS One. 2012 Apr 19;7(4):e35537. doi: 10.1371/journal.pone.0035537 (PMC3334904; doi:10.1371/journal.pone.0035537)
Supplement: Figure S4 — Molecular brightness (CPSM) of eGFP which was transiently expressed in HeLa S3 cells and from HeLa S3 cells stably expressing eGFP-Cdc45 in different cell cycle stages, in UV treated and Asynchronous cells. Error bars correspond to standard deviation from at least 15 cells. (DOC) [file pone.0035537.s004.doc]

**Figure S4**: Molecular brightness (CPSM) of eGFP and HeLa S3 cells stably expressing eGFP-Cdc45 in different cell cycle stages, in UV treated and Asynchronous cells. Error bars correspond to standard deviation from at least 15 cells.
