# Supplementary material for: Cell Cycle-Dependent Mobility of Cdc45 Determined in vivo by Fluorescence Correlation Spectroscopy
Source: PLoS One. 2012 Apr 19;7(4):e35537. doi: 10.1371/journal.pone.0035537 (PMC3334904; doi:10.1371/journal.pone.0035537)
Supplement: Table S1 — Single-component anomalous diffusion model fit to eGFP, eGFP-Cdc45 in different cell cycle stages and UVC treatment. (DOC) [file pone.0035537.s005.doc]

**Table S1**: Single-component anomalous diffusion model fit to eGFP, eGFP-Cdc45 in different cell cycle stages and UVC treatment.

|  | *D* ± SD (μm^2^s^-1^) | Anomality (α) ± SD |
| --- | --- | --- |
| eGFP | 71.6 ± 16.1 | 0.79 ± 0.12 |
| eGFP-Cdc45 | 36.2 ± 10.2 | 0.72 ± 0.1 |
| eGFP-Cdc45  G1/S | 38.6 ± 9.4 | 0.7 ± 0.1 |
| eGFP-Cdc45  S phase | 31 ± 13.0 | 0.7 ± 0.1 |
| eGFP-Cdc45  UVC | 30.3 ± 13.0 | 0.69 ± 0.08 |

SD: standard deviations from at least 15 cells.
