# Supplementary material for: Cell Cycle-Dependent Mobility of Cdc45 Determined in vivo by Fluorescence Correlation Spectroscopy
Source: PLoS One. 2012 Apr 19;7(4):e35537. doi: 10.1371/journal.pone.0035537 (PMC3334904; doi:10.1371/journal.pone.0035537)
Supplement: Table S2 — Two-component anomalous diffusion model fit to eGFP, eGFP-Cdc45 in different cell cycle stages and following UVC treatment. (DOC) [file pone.0035537.s006.doc]

**Table S2**: Two-component anomalous diffusion model fit to eGFP, eGFP-Cdc45 in different cell cycle stages and following UVC treatment.

|  | *D*1 ± SD (μm^2^s^-1^) | r1 ± SD | α1 ± SD | *D*2 ± SD (μm^2^s^-1^) | r2 ± SD | α2 ± SD |
| --- | --- | --- | --- | --- | --- | --- |
| eGFP | 51.5 ± 19.0 | 0.52 ± 0.16 | 1.17 ± 0.3 | 265 ± 135 | 0.48 ± 0.26 | 0.56 ± 0.5 |
| eGFP-Cdc45 | 10.0 ± 3.0 | 0.42 ± 0.13 | 0.88 ± 0.3 | 127 ± 64 | 0.58 ± 0.14 | 0.93 ± 0.4 |
| eGFP-Cdc45  G1/S | 11.0 ± 3.0 | 0.48 ± 0.2 | 0.6 ± 0.3 | 68.7 ± 27.6 | 0.52 ± 0.26 | 1.04 ± 0.26 |
| eGFP-Cdc45  S phase | 5.1 ± 2.1 | 0.35 ± 0.16 | 0.89 ± 0.25 | 66 ± 16 | 0.65 ± 0.16 | 1.03 ± 0.35 |
| eGFP-Cdc45  UVC | 10.4 ± 4.0 | 0.44 ± 0.23 | 0.86 ± 0.3 | 80.6 ± 60 | 0.55 ± 0.23 | 0.78 ± 0.66 |

SD: standard deviations from at least 15 cells.
